# Efficacy of LigaSure Versus Harmonic Devices in Laparoscopic Sleeve Gastrectomy: A Systematic Review and Meta-Analysis

**DOI:** 10.7759/cureus.57478

**Published:** 2024-04-02

**Authors:** Abdullah M Alharran, Yaqoub Y Alenezi, Sabri M Hammoud, Bandar Alshammari, Mohammed Alrashidi, Fajer B Alyaqout, Abdulhadi Almarri, Yousef M Alharran, Mohammed H Alazemi, Fahad Allafi, Khaled Ahmad Al Sadder

**Affiliations:** 1 Medicine and Surgery, College of Medicine, Arabian Gulf University, Manama, BHR; 2 General Surgery, Jaber Alahmad Hospital, Kuwait City, KWT; 3 Medicine and Surgery, Faculty of Medicine, University of Jordan, Amman, JOR; 4 Medicine and Surgery, Faculty of Medicine, Alexandria University, Alexandria, EGY; 5 General Surgery, Jahra Hospital, Jahra, KWT; 6 General Surgery, Ministry of Health, Kuwait City, KWT

**Keywords:** sleeve gastrectomy, laparoscopic sleeve gastrectomy, systematic review and meta-analysis, gastrectomy, harmonic, ligasure

## Abstract

Our goal is to conduct a thorough systematic review and meta-analysis of comparative studies to evaluate the efficacy of LigaSure (Valleylab, Boulder, CO) compared with Harmonic (Ethicon Endo-Surgery, Inc., Cincinnati, OH) devices in patients undergoing laparoscopic sleeve gastrectomy (LSG). Our search strategy, from inception until March 1, 2024, involved multiple databases, including the Cochrane Controlled Register of Trials (CENTRAL), Web of Science (WOS), PubMed, Scopus, and Google Scholar. We evaluated randomized clinical trials using the Cochrane Risk of Bias tool for randomized trials (RoB-2) tool and non-randomized studies using the Risk of Bias In Non-randomized Studies for Interventions (ROBINS-I) tool. The primary outcomes assessed were operative time, mean length of hospital stay, and the rates of intraoperative complications such as bleeding, organ injury, leakage, and hematoma formation. Additionally, we collected data on postoperative complications, including bleeding, abscess formation, leakage, fever (>38°C), and reoperation rates. Data were analyzed using random-effects models and reported as mean difference (MD) or risk ratio (RR) with a 95% confidence interval (CI) using Review Manager software (RevMan, version 5.4 for Windows, The Cochrane Collaboration, 2020). Four studies, comprising two randomized clinical trials (RCTs) and two retrospective cohort studies, involving a total of 692 patients, were included in the analysis. Both the operative time and length of hospital stay did not significantly differ between the LigaSure and Harmonic groups (p>0.05). The pooled analysis also revealed no significant difference between the LigaSure and Harmonic groups in terms of intraoperative and postoperative complications (p>0.05). In conclusion, our systematic review and meta-analysis found no significant statistical or clinical differences between LigaSure and Harmonic devices in terms of operative outcomes and complication rates in patients undergoing LSG.

## Introduction and background

Laparoscopic sleeve gastrectomy (LSG) is now widely accepted as the initial surgical approach for high-risk patients undergoing laparoscopic Roux-en-Y gastric bypass (LRYGB) or biliopancreatic diversion with duodenal switch (BPD-DS) [1,2]. This bariatric procedure has gained popularity due to its relative simplicity and the significant outcomes it has shown over time, both in terms of weight loss and the management of obesity-related comorbidities [3-5]. Despite its reputation as a safe and effective procedure, LSG can be associated with complications during and after surgery, such as bleeding, staple line leaks, and micronutrient deficiencies [6-8].

LigaSure (Valleylab, Boulder, CO) is a handheld electrothermal device designed for sealing vessels up to 7 mm in diameter. It utilizes low voltage and high current to coagulate tissue. This energy application denatures collagen and elastin in vessels and connective tissue, facilitating vessel fusion. LigaSure is equipped with a feedback-controlled response system that automatically stops energy delivery when the seal cycle is finished, ensuring safe operation [9,10].

Harmonic (Ethicon Endo-Surgery, Inc., Cincinnati, OH) utilizes ultrasonic vibration to achieve four distinct tissue effects, including cutting, coaptation, coagulation, and cavitation. This device is capable of transecting and sealing vessels less than 5 mm in diameter, as well as lymphatics. It is noted for its ability to minimize lateral thermal spread when dissecting near critical structures [4,11-13].

Recent studies [10,13-15] have investigated the differences between LigaSure and Harmonic devices in terms of intraoperative and postoperative outcomes among patients undergoing LSG. The best available evidence regarding the efficacy of LigaSure compared with Harmonic has not yet been synthesized. Therefore, our aim is to conduct a comprehensive systematic review and meta-analysis of comparative studies to assess the efficacy of LigaSure compared with Harmonic devices among patients undergoing LSG.

## Review

Methods

This systematic review and meta-analysis of comparative studies adhered closely to the guidelines outlined in the Preferred Reporting Items for Systematic Reviews and Meta-Analyses (PRISMA) statement [3] as well as the Cochrane Handbook for Systematic Reviews and Meta-Analyses of Interventions [16]. As this review is based on published studies, ethical approval is not necessary. Our population, intervention, control, outcomes, and study (PICOS) criteria were as follows: (P) morbidly obese patients undergoing LSG; (I) LigaSure device; (C) Harmonic device; (O) efficacy endpoints; and (S) comparative studies such as randomized clinical trials (RCTs), quasi-randomized clinical trials, and cohort studies. We excluded single-arm studies, studies with unreliable data for extraction and analysis, studies reported only as abstracts or theses, studies with incomplete full texts, case reports, case series, review articles, and studies not published in English.

Data and Study Selection

From the beginning until March 1, 2024, our approach relied on searching multiple databases for relevant studies, including the Cochrane Controlled Register of Trials (CENTRAL), Web of Science (WOS), PubMed, Scopus, and Google Scholar. Our search strategy encompassed terms such as "Laparoscopic Sleeve Gastrectomy," "Sleeve Gastrectomy," "Laparoscopic Sleeve Gastrectom*," "Gastrectom*," "LSG," combined with terms like "Harmonic," "Harmonic Scalpel," "Ultrasonic Scalpel," "Ultrasonic Harmonic Scalpel," "Harmonic Ace," and also "ligasure," "Ligasure™," "Ligasure device," "ligasure scalpel." Detailed search strategies for each database are provided in Table 1.

**Table 1:** The exact literature search strategy used in every database CENTRAL: Cochrane Controlled Register of Trials

The exact literature search strategy used in every database
[PubMed] All Field: (“Laparoscopic Sleeve Gastrectomy” OR “Sleeve Gastrectomy” OR “Laparoscopic Sleeve Gastrectom*” OR “Gastrectom*” OR “LSG”) AND (Harmonic OR “Harmonic Scalpel” OR “Ultrasonic Scalpel” OR “Ultrasonic Harmonic Scalpel” OR “Harmonic Ace”) AND (“ligasure” OR “Ligasure™” OR “Ligasure device” OR “ligasure scalpel”).
[Scopus] Article title, Abstract, Keywords: (“Laparoscopic Sleeve Gastrectomy” OR “Sleeve Gastrectomy” OR “Laparoscopic Sleeve Gastrectom*” OR “Gastrectom*” OR “LSG”) AND (Harmonic OR “Harmonic Scalpel” OR “Ultrasonic Scalpel” OR “Ultrasonic Harmonic Scalpel” OR “Harmonic Ace”) AND (“ligasure” OR “Ligasure™” OR “Ligasure device” OR “ligasure scalpel”).
[Web of Science] All Fields: (“Laparoscopic Sleeve Gastrectomy” OR “Sleeve Gastrectomy” OR “Laparoscopic Sleeve Gastrectom*” OR “Gastrectom*” OR “LSG”) AND (Harmonic OR “Harmonic Scalpel” OR “Ultrasonic Scalpel” OR “Ultrasonic Harmonic Scalpel” OR “Harmonic Ace”) AND (“ligasure” OR “Ligasure™” OR “Ligasure device” OR “ligasure scalpel”).
[Cochrane CENTRAL] Title Abstract Keyword: (“Laparoscopic Sleeve Gastrectomy” OR “Sleeve Gastrectomy” OR “Laparoscopic Sleeve Gastrectom*” OR “Gastrectom*” OR “LSG”) AND (Harmonic OR “Harmonic Scalpel” OR “Ultrasonic Scalpel” OR “Ultrasonic Harmonic Scalpel” OR “Harmonic Ace”) AND (“ligasure” OR “Ligasure™” OR “Ligasure device” OR “ligasure scalpel”).
[Google Scholar] All Fields: (“Laparoscopic Sleeve Gastrectomy” OR “Sleeve Gastrectomy” OR “Laparoscopic Sleeve Gastrectom*” OR “Gastrectom*” OR “LSG”) AND (Harmonic OR “Harmonic Scalpel” OR “Ultrasonic Scalpel” OR “Ultrasonic Harmonic Scalpel” OR “Harmonic Ace”) AND (“ligasure” OR “Ligasure™” OR “Ligasure device” OR “ligasure scalpel”).

To expand our literature search, we reviewed the reference lists of eligible studies and recent reviews to identify any potentially overlooked relevant studies. The process of selecting studies involved removing duplicate citations, screening titles and abstracts, and then reviewing the full texts of potentially relevant citations. Two co-authors independently carried out the search strategy and study selection, with any discrepancies resolved through consultation with the principal investigator.

Risk of Bias Assessment

We utilized the Risk of Bias in Non-Randomized Studies for Interventions (ROBINS-I) [17] tool to assess bias in the cohort and non-randomized studies. This tool comprises seven domains: confounding, selection of interventions, classification of interventions, deviations from intended interventions, missing data, measurement of outcomes, and selection of the reported result. Additionally, we employed the Cochrane Risk of Bias tool for randomized trials (RoB-2) [18] to assess bias for RCTs in the randomization process, deviations from intended interventions, missing outcome data, measurement of the outcome, selection of the reported result, and other forms of bias. The judgments of the authors were categorized as "low risk," "high risk," or "some concerns" of bias. Two authors independently conducted the quality assessment, resolving any disagreements through discussion.

Data Collection and Review Outcomes

Data collection proceeded in three stages. Initially, we compiled a summary list of citation features and characteristics, including study ID, country, duration, total sample size, and number of study arms. Next, we gathered data on patient demographics, such as sample size, age, sex, and morbidity indicators like hypertension and diabetes, for both intervention and control groups. Lastly, we collected data on efficacy outcomes, including operative time, mean length of hospital stay, and rates of intraoperative complications such as bleeding, organ injury, leakage, and hematoma formation. Additionally, we collected data on postoperative complications, including bleeding, abscess formation, leakage, fever (>38°C), and reoperation rates.

Meta-Analysis 

We utilized the Review Manager program (RevMan, version 5.4 for Windows, The Cochrane Collaboration, 2020) for the pooled analysis. Binary and continuous outcomes were collected under the random-effects model to calculate the odds ratio (OR) and mean difference (MD), respectively, along with 95% confidence intervals (CI). The analysis relied on the inverse variance and Mantel-Haenszel techniques. Heterogeneity was assessed using chi-square tests, with substantial heterogeneity noted when the chi-square test had a p-value <0.05. Statistical significance for all endpoints was defined as a p-value <0.05. Due to the small number of studies (less than 10) included in our analysis, the assessment of publication bias is not reliable. Therefore, we were unable to evaluate the presence of publication bias using Egger's test for funnel plot asymmetry in our study [16].

Results

Summary of the Literature Search

**Figure 1:**
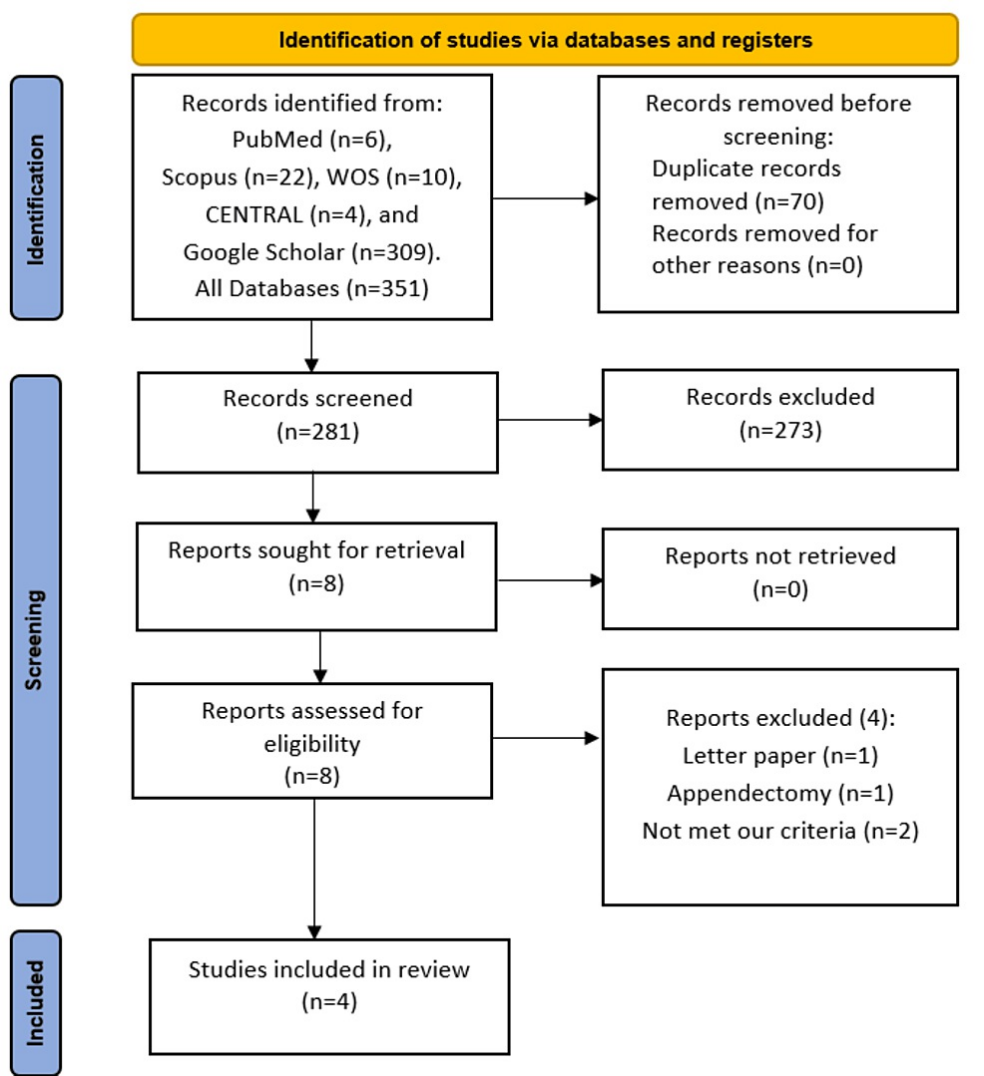
PRISMA flow diagram outlining the study selection process PRISMA: Preferred Reporting Items for Systematic Reviews and Meta-Analyses; CENTRAL: Cochrane Controlled Register of Trials; WOS: Web of Science

After removing 70 duplicate citations, our initial search yielded 281 articles. Subsequently, during the title and abstract screening, 273 citations were excluded. Finally, after reviewing the full texts, four studies [10,13-15] met our PICOS criteria. The PRISMA diagram illustrating our search process is presented in Figure 1.

Summary of the Included Studies

We included four studies, consisting of two RCTs and two retrospective cohort studies, involving a total of 692 patients. These studies were conducted in Egypt, Turkey, Greece, and Italy. Detailed information for each study is provided in Tables 2-3.

**Table 2:** Summary of the included studies [10,13-15]

Study ID	Country	Design	Duration	Sample size	Study arms		Included patients
					Intervention	Control	
Makal, 2020 [15]	Turkey	Retrospective cohort study	March 2015 and April 2020	n=139	LigaSure	Harmonic	Morbidly obese patients undergoing laparoscopic sleeve gastrectomy
Sabry et al., 2018 [14]	Egypt	Randomized clinical trial	Not reported	n=37	LigaSure	Harmonic	Morbidly obese patients undergoing laparoscopic sleeve gastrectomy
Tsamis et al., 2015 [10]	Greece	Randomized clinical trial	Not reported	n=94	LigaSure	Harmonic	Morbidly obese patients undergoing laparoscopic sleeve gastrectomy
Velotti et al., 2019 [13]	Italy	Retrospective cohort study	January 2009 and December 2017	n=422	LigaSure	Harmonic	Morbidly obese patients undergoing laparoscopic sleeve gastrectomy

**Table 3:** Baseline characteristics of the included studies [10,13-15]

Study ID	Group	Sample size, n	Age (years)	Sex, n	BMI (kg/m^2^)	Diabetes, n	Hypertension, n
				(Male/Female)			
Makal, 2020 [15]	LigaSure	n=82	38 (16-69)	(18/64)	40 (30-60)	9	12
	Harmonic	n=54	39 (16-62)	(14/40)	41 (32-59)	6	8
Sabry et al., 2018 [14]	LigaSure	n=20	36.65 ±10.10	(3/17)	50.55±4.63	Not reported	Not reported
	Harmonic	n=17	34.88 ±8.07	(5/12)	49.64±5.74	Not reported	Not reported
Tsamis et al., 2015 [10]	LigaSure	n=43	39 ±16	(17/26)	45.2±8	Not reported	Not reported
	Harmonic	n=51	32.5 ±17	(18/33)	45.7 ±6.07	Not reported	Not reported
Velotti et al., 2019 [13]	LigaSure	n=225	42.6 ±10.73	(50/175)	47.2±6.11	81	67
	Harmonic	n=197	41.2 ±8.26	(58/139)	47.7±5.23	77	75

Summary of the Risk of Bias Assessment

**Figure 2:**
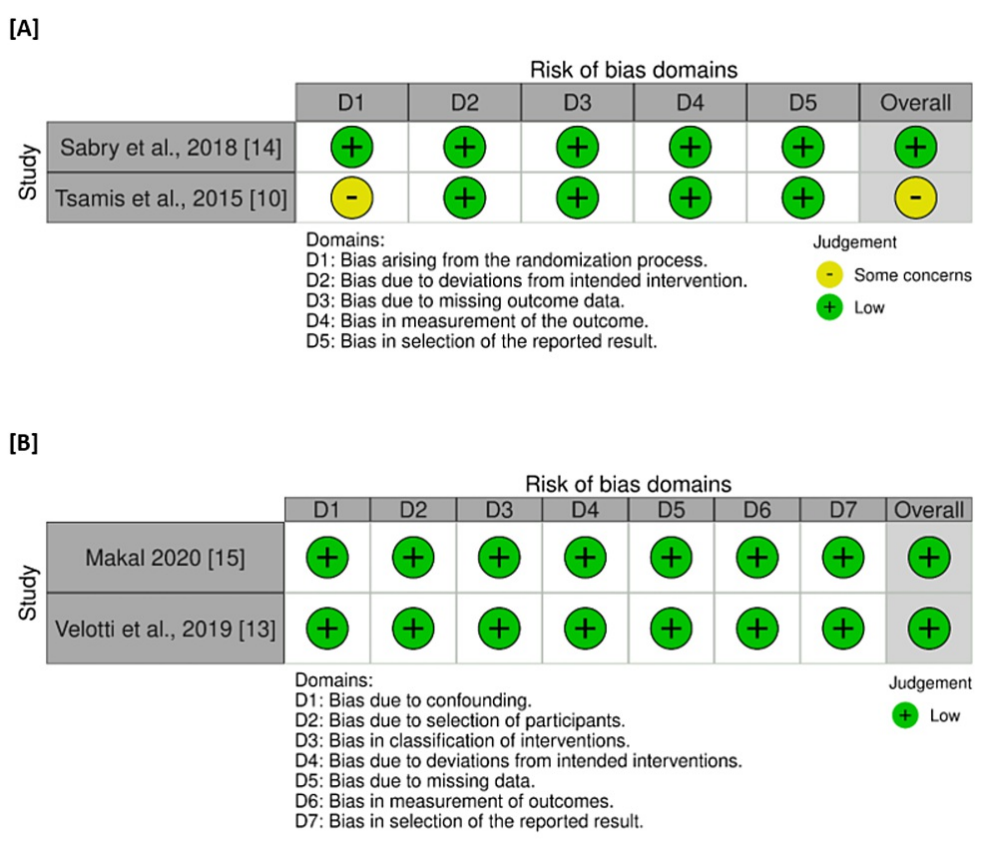
Risk of bias graphs of (A) randomized clinical trials, and (B) retrospective cohort studies [10,13-15]

Three out of the four studies were assessed as having a low risk of bias. However, Tsamis et al. [10] were evaluated as having some concerns regarding the randomization process domain. The reason was that they did not provide sufficient information regarding the randomization process and allocation concealment for patient enrollment and distribution. Figure 2 depicts the risk of bias graph for each study.

Operative Time (Minutes) and Length of Hospital Stay (Days)

**Figure 3:**
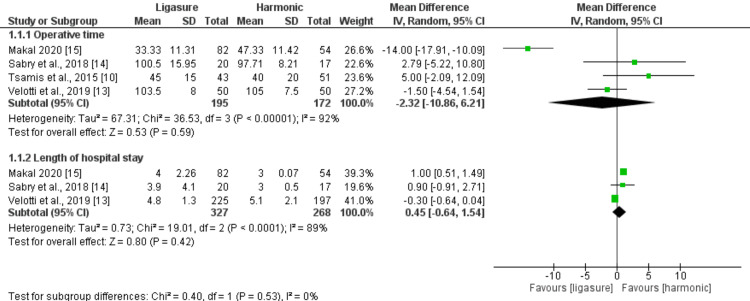
Meta-analysis of operative time (minutes) and length of hospital stay (days) [10,13-15]

Both the operative time and length of hospital stay did not significantly differ between the LigaSure and Harmonic groups (n = 367 patients, MD: -2.32, 95% CI (-10.86, 6.21), p = 0.59) and (n = 595 patients, MD: 0.45, 95% CI (-0.64, 1.54), p = 0.42), respectively. All the pooled analyses were heterogeneous (I2> 50%). Figure 3 illustrates the summary of the analysis.

Intraoperative Complications (%)

**Figure 4:**
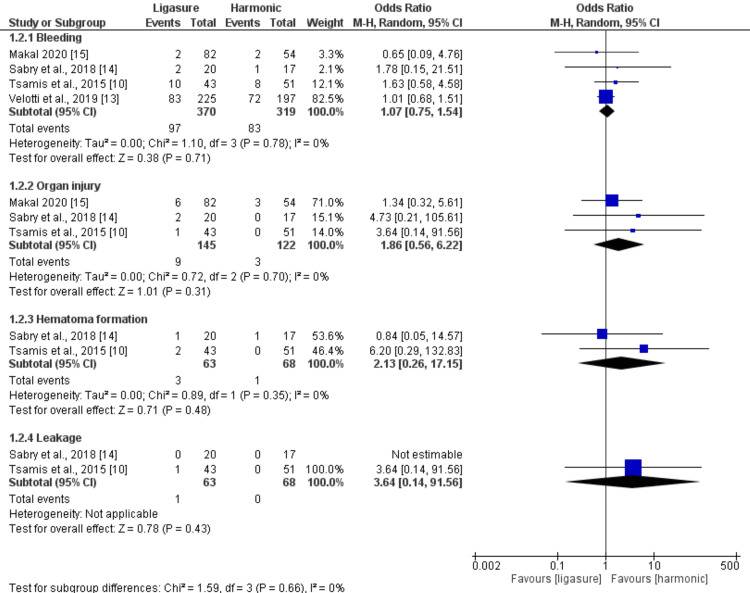
Meta-analysis of the rate of intraoperative complications (%) [10,13-15]

The pooled analysis revealed no significant difference between the LigaSure and Harmonic groups regarding the rate of bleeding (n = 689 patients, OR: 1.07, 95% CI (0.75, 1.54), p = 0.71), the rate of organ injury (n = 267 patients, OR: 1.86, 95% CI (0.56, 6.22), p = 0.31), the rate of hematoma formation (n = 131 patients, OR: 2.13, 95% CI (0.26, 17.15), p = 0.48), and the rate of leakage (n = 131 patients, OR: 3.64, 95% CI (0.14, 91.56), p = 0.43). All the pooled analyses were homogeneous (I2<50%). Figure 4 illustrates the summary of the analysis.

Postoperative Complications (%)

**Figure 5:**
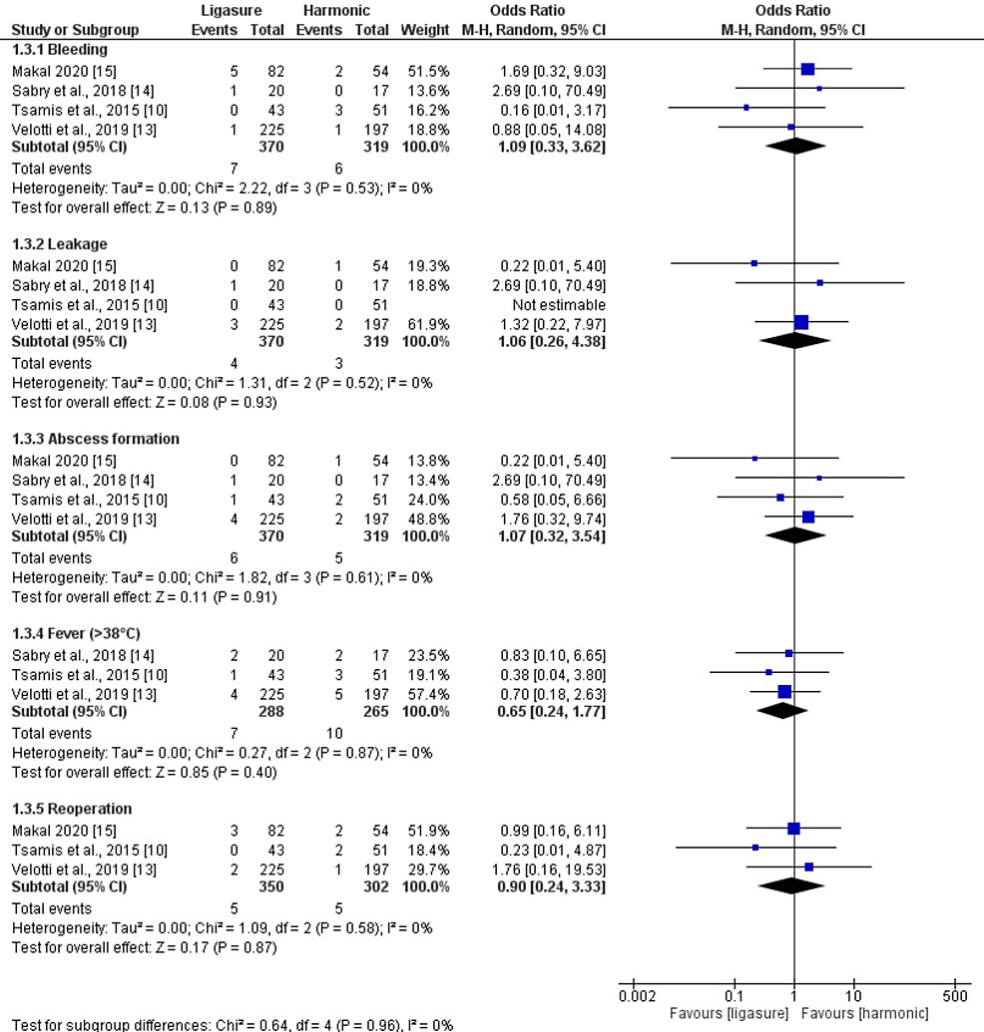
Meta-analysis of the rate of postoperative complications (%) [10,13-15]

The pooled analysis revealed no significant difference between the LigaSure and Harmonic groups regarding the rate of bleeding (n = 689 patients, OR: 1.09, 95% CI (0.33, 3.62), p = 0.89), the rate of leakage (n = 689 patients, OR: 1.06, 95% CI (0.26, 4.38), p = 0.93), the rate of abscess formation (n = 689 patients, OR: 1.07, 95% CI (0.32, 3.54], p = 0.91), the rate of fever (n = 553 patients, OR: 0.64, 95% CI (0.24, 1.77), p = 0.40), and the rate of reoperation (n = 652 patients, OR: 0.90, 95% CI (0.24, 3.33), p = 0.87). All the pooled analyses were homogeneous (I2<50%). Figure 5 illustrates the summary of the analysis.

Discussion

The findings of this systematic review and meta-analysis suggest that there is no significant difference in terms of operative outcomes and complication rates between LigaSure and Harmonic devices in patients undergoing LSG. This is an important finding, as it indicates that both devices can be considered equally effective and safe for use in LSG procedures. The lack of significant differences in operative time and length of hospital stay suggests that both devices are comparable in terms of efficiency and postoperative recovery. This is particularly relevant in the context of bariatric surgery, where minimizing the operative time and duration of hospital stay can lead to improved patient outcomes and reduced healthcare costs.

Regarding intraoperative and postoperative complications, the results of this study provide reassurance that both LigaSure and Harmonic devices are associated with similar rates of adverse events such as bleeding, organ injury, hematoma formation, leakage, and reoperation. This suggests that neither device has a clear advantage over the other in terms of its safety profile. Clinicians can therefore choose between these devices based on factors such as cost, availability, and surgeon preference without compromising patient outcomes.

Despite the overall low risk of bias in the included studies, it is important to note that one study did not provide sufficient information regarding the randomized process and allocation concealment methods. This highlights the need for future studies in this area to adhere to rigorous methodological standards to ensure the validity and reliability of their findings. Additionally, further research is warranted to assess long-term outcomes such as weight loss maintenance and quality of life following LSG using these devices.

Complications in LSG are influenced by several factors, including the surgeon's skill, patient characteristics (such as comorbidities, medications, and genetics), technical resources, and the quality of surgical materials. The aim of advancing surgical techniques is to reduce these complications [15]. Among the risks, bleeding and leakage are especially worrisome in LSG. To manage bleeding, various techniques like energy devices, electrocautery, clips, vascular staplers, and sutures are used. While electrocautery is easy to use, its effectiveness is limited in achieving proper hemostasis [15]. Additionally, it generates significant heat, which can lead to thermal injury, especially in bowel walls. LigaSure and Harmonic devices are the most commonly used energy devices in laparoscopic surgeries.

Numerous studies have compared the effectiveness of LigaSure and Harmonic devices in various surgical fields, such as endocrine, colorectal, and gynecological surgery [19-22]. However, the best available evidence, particularly in laparoscopic procedures, is limited. Previous studies have indicated comparable advantages to using both devices. Campagnacci et al. [23] found no significant difference in the duration of colorectal surgery between the two devices, but there was less bleeding with LigaSure. Similarly, Yavuz et al. [24] did not detect any statistical difference in a randomized trial involving 24 cases of laparoscopic appendicectomy. Rimonda et al. [25] analyzed results from 140 patients undergoing various colorectal procedures and concluded that both LigaSure and Harmonic are useful and safe instruments for laparoscopic colorectal surgery, with no significant difference in terms of intraoperative or postoperative morbidity or operative time.

Strengths and limitations

This meta-analysis represents the first comprehensive investigation comparing the efficacy of LigaSure versus Harmonic devices in LSG. The inclusion of multiple outcomes provides a detailed assessment of the potential superiority of one technique over the other. However, the study has several limitations. Firstly, only four studies with mixed study designs and relatively small sample sizes were included. Additionally, there was a significant level of heterogeneity observed in some outcomes across the included studies. Furthermore, discrepancies in the reported methodologies were noted among the included studies.

## Conclusions

In conclusion, based on the available evidence, our analysis suggests that there are no significant differences between LigaSure and Harmonic devices in terms of operative time, length of hospital stay, rates of intraoperative complications (e.g., bleeding, organ injury, hematoma formation, and leakage), or postoperative complications (e.g., bleeding, abscess formation, leakage, fever (>38°C), and reoperation rates) among patients undergoing LSG.
